# *Pseudomonas* spp. Enriched in Endophytic Community of Healthy Cotton Plants Inhibit Cotton *Verticillium* Wilt

**DOI:** 10.3389/fmicb.2022.906732

**Published:** 2022-07-18

**Authors:** Qingchao Zeng, Xiaowu Man, Yucheng Dai, Haiyang Liu

**Affiliations:** ^1^Beijing Advanced Innovation Center for Tree Breeding by Molecular Design, Beijing Forestry University, Beijing, China; ^2^Institute of Plant Protection, Xinjiang Academy of Agricultural Sciences, Ürümqi, China

**Keywords:** cotton *Verticillium* wilt, endophytic, bacterial community, microbiome assembly, beneficial microbe

## Abstract

The plant microbiome plays a fundamental role in plant growth and health. However, detailed information regarding the plant endophytic microbiome during the infection period of a pathogen is largely unknown. Here, we investigated the microbial community of healthy and diseased cotton plants and the root exudate profiles of susceptible and resistant cultivars utilizing high-throughput sequencing and metabolomics. The results showed that the pathogen infection reduced bacterial diversity and significantly affected the bacterial community composition. The microbiome assembly is shaped predominantly by cultivars. The endophytic microbiome of the infected plants showed greater complexity than the healthy plants in network analysis. The results displayed that a total of 76 compounds were significantly different in the two groups, with 18 compounds showing a higher relative abundance in the resistant cultivars and 58 compounds in the susceptible cultivars. Pathway enrichment analysis showed that pathways related to plant hormone signal transduction, biosynthesis of various secondary metabolites, and biosynthesis and metabolism of amino acids were prominently altered. We also demonstrate that plants inoculated with *Pseudomonas* sp. strains showed increased resistance to the cotton *Verticillium* wilt compared with the control plants in pot experiments. Overall, it showed that the pathogen infection affected the community composition, and healthy plants displayed an enriched beneficial microbiome to combat the plant disease. These findings significantly advance our understanding of the endophytic microbiome assembly under the pathogen infection and develop microbiome-based solutions for sustainable crop production systems.

## Introduction

Plants host enormous diverse communities of microorganisms, and the plant microbiome, which equips the host plant with additional gene pools, is often referred to as the second plant genome or extended genome (Berendsen et al., 2012; Vandenkoornhuyse et al., 2015; Chen et al., 2019). The plant microbiome coevolved with the hosts and profoundly impacts a range of plant functions and fitness parameters, such as disease suppression, stress tolerance, and growth promotion (Carrion et al., 2019; Liu et al., 2021a; Zhuang et al., 2021). Therefore, engineering the plant microbiome is increasingly considered as the goal of improving plant health and productivity. In recent years, more and more studies found that plants recruited certain beneficial microorganisms to resist the invasion of pathogens. For example, the resistant tomato variety Hawaii 7996 could recruit members of *Flavobacterium* to suppress plant disease for the rhizosphere microbiome. *Astragalus mongholicus* recruited some beneficial bacteria, such as *Pseudomonas* and *Flavobacterium*, to the rhizosphere and roots under the fungal pathogen infection (Kwak et al., 2018; Li et al., 2021). Therefore, understanding how plant modulates their microbiome under pathogen infection is the prerequisite for manipulating plant microbiome to enhance plant health.

The plant microbiome is influenced by multiple biotic and abiotic factors, such as genotype, soil properties, plant development stage, and pathogen infection (Carrion et al., 2019; Hu et al., 2020; Liu et al., 2021b; Zhuang et al., 2021). Among them, pathogen invasion is one of the most important biotic stress factors that affect the microbiome assembly. The microbial diversity and composition were altered in the decaying sugar beet compared to that observed in the healthy plants (Kusstatscher et al., 2019). Meanwhile, the microbiome plays a key role in protecting plants from pathogens. Accumulating research on sugar beet, tomato, *Arabidopsis thaliana*, and wheat has shown that plants could recruit beneficial microbes for protecting plants (Rudrappa et al., 2008; Carrion et al., 2019; Lee et al., 2021; Liu et al., 2021a). The phyllosphere microbiome could protect *Arabidopsis* plants against the fungal pathogens (Ritpitakphong et al., 2016). Rudrappa et al. displayed that *A. thaliana* leaf infections by *P. syringae* induced root exudation of malic acid that selectively recruited the beneficial *Bacillus* strain FB17 (Rudrappa et al., 2008). The mentioned results also showed that the below- and aboveground microbiome of plants have a close relationship. Furthermore, biostimulators and organic fertilizers are known to affect the plant microbiome and enrich beneficial microorganisms. The application of organic fertilizer showed that it improved resistance against plant pathogens and influenced the structure and function of the resident soil microbiome (Tao C. et al., 2020). Meantime, it has been reported that bacteriophages act as an alternative to pesticides to control pathogens and found that it did not affect the existing rhizosphere microbiome and enriched bacterial species which were antagonistic toward plant pathogens (Wang et al., 2019). Moreover, the glutamic acid modulated the composition of the microbiome community and enriched the *Streptomyces* populations to control the disease caused by *Botrytis* and *Fusarium* (Kim et al., 2021). Other studies have displayed that the susceptible cultivars tend to form disease-suppressive soils more easily than the resistant cultivars. Interestingly, the root secretion patterns with the same plant species were different, which caused variation in the composition of the microbial community. For example, the susceptible cucumber secreted root exudates, such as organic acid, to assemble beneficial microorganisms (Weller et al., 2002; Wen et al., 2020). It reported that the host selection pressure increased from soil to endophytes (Xiong et al., 2021). To date, most studies focused on the belowground (rhizosphere and soil microbiome) and phyllosphere microbiome. The detailed information on the plant endophytic microbiome during the infection period of a pathogen is largely unknown.

Cotton (*Gossypium* spp.) is one of the most important crops in the world due to its economic value in textile fiber, feed, foodstuff, oil, and biofuel products (Wu et al., 2021). Cotton plantation in Xinjiang is one of the most important cotton production regions worldwide and plays an important role in the economy. However, cotton *Verticillium* wilt, a devastating disease of cotton, is caused by the soil-borne plant fungus *Verticillium dahliae* Kleb and affected cotton production and quality. Long-term monocropping leads to a severe incidence of cotton *Verticillium* wilt. Many genera, including *Bacillus*, *Pseudomonas*, *Streptomyces*, and *Enterobacter*, have documented biocontrol activities against cotton *Verticillium* wilt (Li et al., 2013; Xue et al., 2013; Tao X. et al., 2020; Liu et al., 2021b). The underlying mechanisms are known to be involved in biological control, including reduced germination of inoculum, plant growth promotion, induced resistance, and antibiotic production (Deketelaere et al., 2017). The endophyte isolated from the plant’s interior tissue improves plant resistance to pathogens. These traits make endophytes the potential natural resources for biological control. Endophytes are beneficial to the plants, including growth promotion and biological control against plant pathogens (Erdogan and Benlioglu, 2010). The endophyte *P. fluorescens* PICF7 isolated from the roots of olive trees displayed the suppression of *Verticillium* wilt. The colonization by the endophyte in roots could trigger defense responses and compete for the niche with the pathogen (Prieto et al., 2009; Gómez-Lama Cabanás et al., 2014). Furthermore, *Pseudomonas* plays a vital role in the plant microbiome, particularly in plant growth promotion and disease suppression. For example, the *B. velezensis* SQR9 induced the enrichment of *Pseudomonas* spp. in the cucumber rhizosphere to promote plant growth based on the microbiome analysis (Sun et al., 2022). Meanwhile, *Pseudomonas* was the key plant growth-promoting rhizosphere of garlic and has the ability to promote plant growth (Zhuang et al., 2021). It has also been reported that *Pseudomonas*, *Sphingomonas*, and *Burkholderia* showed higher abundances in the field with less incidence of disease (Zhang et al., 2022). It is not clear why the healthy plants selected specific endophytic bacterial genus to inhibit the phytopathogens. Here, we used two types of cotton cultivars with different phenotypes under the pathogen infection. The objectives of this study were to (1) investigate the endophytic bacterial communities from healthy and diseased plants infected with cotton *Verticillium* wilt for susceptible and resistant cultivars and (2) determine whether enrichment with microorganisms in healthy cotton plants could confer disease prevention. To address the above questions, we first examined the dynamics of endophytic bacterial communities of cotton plants with and without disease symptoms for two cultivars. Second, we isolated two strains of *Pseudomonas* sp. that were enriched in healthy plants and checked the effect of inhibition on cotton *Verticillium* wilt. In addition, we analyzed the root metabolism of susceptible and resistant cultivars. The overall results advance our understanding of the microbiome assembly under the influence of pathogen infection and develop microbiome-based solutions for sustainable crop production systems.

## Materials and Methods

### Experimental Design and Sample Collection

The experiment was designed in the main cotton production fields of Korla (41°45’2.39”N, 85°48’4.69”E), Xinjiang province, Northwest China. The site is located in the temperate continental climate zone, with the same annual mean temperature of 11°C and annual mean precipitation of 58.6 mm. The cotton cultivars used in this study were Zhongzhimian No. 2 (resistant cultivar) and Junmian No. 1 (susceptible cultivar). The cotton plants were sampled in June 2017. Briefly, the 2,500 m^2^ cotton field was selected as the experiment area, and 400 kg of *V. dahliae* was added when the cotton was planted in 2014 and 2015. The disease symptom was observed in 2014. Cotton plants in the experimental area that displayed no wilt or disease symptoms were classified as healthy plants, while the plants in the experimental area that showed wilt or obvious disease symptoms were classified as diseased plants (Supplementary Figure 1). Three replicates of healthy and diseased cotton plants were collected from three adjacent plots, and each replicate consisted of a composite sample obtained by mixing five individual samples. The leaves of the cotton plant were discarded, and the stem was cut into small parts and stored in a 50 mL tube. All the samples were kept in cold conditions and later transferred to the laboratory and stored at –80°C before further processing. The soil type in the designed field was loam sandy soil. The pH, total nitrogen, total phosphorus, total potassium concentration, and organic matter were 7.98, 0.42 g/kg, 0.67 g/kg, 16.51 g/kg, and 8.94 g/kg, respectively.

### DNA Extraction and Bacterial 16S rRNA Gene Amplification

For microbial DNA extraction from the endosphere, the plant stem materials were placed in sterile tubes containing phosphate-buffered saline buffer (137 mM NaCl, 2.7 mM KCl, 10 mM Na2HPO4, and 2 mM KH2PO4) and sonicated at 50 kHz for 90 s. This procedure was repeated three times. Then, the plant stem material was rinsed with 75% ethanol for 2 min, then the sample was washed with 1% NaCIO for 1 min, and finally washed again with sterile H_2_O for three times, to ensure surface sterilization. Then, the stem material was ground using sterile mortar with liquid nitrogen to extract DNA. Total DNA was extracted from the plant using Plant DNA mini kit (Omega, Beijing, China) according to the manufacturer’s protocol. The quality and quantity of DNA were measured using NanoDrop 2000 spectrophotometer (Thermo Scientific, Waltham, MA, United States). For the 16S rRNA gene library, the V3-V4 region was amplified using the universal primers 338F (ACTCCTACGGGAGGCAGCA) and 806R (GGACTACHVGGGTWTCTAAT) (Huws et al., 2007). The PCR reaction was performed in 10 μL volume, containing 0.3 μL of both primers (10 μM), 50 ng of template DNA, 5 μL of KOD FX Neo buffer, and 0.2 μL of KOD FX Neo (TOYOBO, Shanghai, China). Amplification was carried out with the following PCR program: initial denaturation at 95°C for 5 min, followed by 25 cycles of 95°C for 30 s, 50°C for 30 s, and 72°C for 40 s, and final elongation at 72°C for 7 min. The library quality was assessed using the Qubit 2.0 Fluorometer (Thermo Scientific) and Agilent Bioanalyzer 2100 system. Finally, the library was sequenced on an Illumina Hiseq 2500 platform at Biomarker Co., Ltd (Beijing, China), and 250 bp paired-end reads were generated.

### Bioinformatics Analysis

Raw sequences were split according to their unique barcodes, and the adaptors and primer sequences were trimmed using their own script from the company. Paired-end sequences were merged using FLASH software 1.2.11 with the default parameters (Magoc and Salzberg, 2011). Then, the amplicon data were analyzed using a combination of the VSEARCH v2.13.3 and QIIME 1.9.1. software (Caporaso et al., 2010; Zgadzaj et al., 2016). The resultant sequences were quality-filtered, and singletons were removed in VSEARCH. The sequence reads were then clustered into OTUs at 97% similarity level. Chimeric sequences that were identified using the reference-based chimera checking methods were removed from the data. Representative sequences were classified using the blast algorithm with the SILVA (v.13.2) reference database in QIIME (Quast et al., 2013). Mitochondrial and chloroplastic DNA sequences, and OTUs with a total relative abundance of < 0.00001 in all of the samples were discarded. The raw sequencing data have been submitted to the Sequence Read Archive under accession number PRJNA820148.

### Root Exudate Collection and UHPLC-QE-MS Detection

The sample was prepared as previously reported, with little modification (Wen et al., 2020). For extraction, 50 mg of sample was taken and placed in an EP tube, and then 1,000 μL of extraction solvent containing an internal target was added (*V*_*methanol*_: *V*_*acetonitrile*_: *V*_*water*_ = 2:2:1, which was kept at –20°C before extraction). The mixtures were homogenized in a ball mill for 4 min at 45 Hz, and then ultrasound treated for 5 min (incubated in ice water). After homogenization for three times, the sample was incubated for 1 h at –20°C to precipitate the proteins, and then centrifuged at 4°C and 12,000 rpm for 15 min. Next, the supernatant (500 μL) was transferred into a fresh tube, the extracts were dried in a vacuum concentrator without heating, and 100 μL of extraction solvent was added (*V*_*acetonitrile*_: *V*_*water*_ = 1:1) for reconstitution. The sample was vortexed for 30 s, sonicated for 10 min (4°C water bath), and centrifuged for 15 min at 12,000 rpm and 4°C. Then, the supernatant (60 μL) was transferred to a fresh 2 mL LC/MS glass vial for the UHPLC-QTOF-MS analysis.

The UHPLC-QTOF-MS analysis was performed using a UHPLC system (1290, Agilent Technologies, Palo Alto, CA, United States) with a UPLC BEH Amide column (1.72 μm, 2.1*100 mm, Waters, Milford, CT, United States) coupled to TripleTOF 6600 (QTOF, AB Sciex, Framingham, MA, United States). The mobile phase consisted of 25 mM NH_4_OAc and 25 mM NH_4_OH in water (pH = 9.75) (A) and acetonitrile (B), and elution was carried out with elution gradient as follows: 0 min, 95% B; 7 min, 65% B; 9 min, 40% B; 9.1 min, 95% B; and 12 min, 95% B, which were delivered at 0.5 mL min^–1^. The injection volume was 2 μL. The Triple TOF mass spectrometer was used due to its ability to acquire MS/MS spectra on an information-dependent basis (IDA) during an LC/MS experiment. In this mode, the acquisition software (Analyst TF 1.7, AB Sciex, Framingham, MA, United States) continuously evaluates the full-scan survey MS data as it collects and triggers the acquisition of MS/MS spectra depending on the preselected criteria. In each cycle, 12 precursor ions whose intensity was greater than 100 were chosen for fragmentation at the collision energy (CE) of 30 V (15 MS/MS events with a product ion accumulation time of 50 ms each). The following settings were used: ion source gas 1 at 60 Psi, ion source gas 2 as 60 Psi, curtain gas at 35 Psi, source temperature at 650°C, and ion spray voltage floating (ISVF) at 5,000 V or –4,000 V in positive or negative modes, respectively.

MS raw data (.wiff) files were converted to the mzXML format using ProteoWizard and processed by R package XCMS (version 3.2). The preprocessing results generated a data matrix that consisted of the retention time (RT), mass-to-charge ratio (m/z) values, and peak intensity. R package CAMERA was used for peak annotation after data processing by XCMS. An in-house MS2 database was applied for the identification of metabolites.

### Statistical Analysis

The OTU table was rarefied to reads (lowest number of 3,616 for bacterial community) for alpha-diversity and was used as a normalization method for beta-diversity analysis. Bacterial alpha-diversity (observed OTUs and Shannon index) values were calculated using QIIME. The significant differences were evaluated with a one-way analysis of variance (ANOVA). Bacterial beta-diversity was estimated according to the Bray-Curtis distance between samples. Permutational multivariate analysis of variance (PERMANOVA) was performed with 1,000 permutations using the adonis function in vegan R package (Xiong et al., 2021). The Venn diagram was drawn using the OECloud tools^1^ to analyze common and specific OTUs for healthy and disease plants of different cultivars. The linear discriminant analysis effect size (LEfSe) was applied (Wilcoxon *P*-value < 0.05 and logarithmic LDA (linear discriminant analysis) score > 2)^2^ to identify the biomarkers of healthy and diseased plants and different cultivars (Xiong et al., 2021). Welch’s test was used to calculate the significance of differences between different healthy conditions using STAMP software (Parks et al., 2014). Differential abundance analysis was performed using EdgeR’s generalized linear model (GLM) approach, and the differential OTUs with false discovery rate-corrected *P*-values < 0.05 were identified as indicator OTUs (Robinson et al., 2010).

The network analysis based on Pearson correlation scores was performed using the CoNet in CYTOSCAPE v3.8 and visualized in GEPHI (Shannon et al., 2003; Xiong et al., 2021). Briefly, only robust (Pearson’s *r* > 0.9 and *r* < –0.9) and statistically significant (*p* < 0.01) correlations were retained for the next step. The ‘benjaminihochberg’ multiple testing correction (*p* < 0.01) was performed at bootstrap step restore network step. The topological indices of the networks were calculated in Gephi. Network modular analysis was conducted in Gephi with default parameters. The network complexity was defined according to previous research, and nodes with high degree (>30) and closeness centrality (>0.6) values were identified as hub nodes in the network (Agler et al., 2016; van der Heijden and Hartmann, 2016; Wagg et al., 2019). Analysis was conducted in R 3.0.6 or GraphPad Prism 7.

To investigate the metabolism of different cultivars, if the metabolite feature is detected in <20% of experimental samples or is detected in <50% of quality control samples, then it is deleted from the data analysis. Then the missing values of raw data were filled up by half of the minimum value. In addition, the internal standard normalization method was employed in this data analysis. Finally, features with a relative standard deviation > 30% should be removed from the subsequent analysis. The resulting three-dimensional data involving the peak number, sample names, and normalized peak area were fed into R package metaX for principal component analysis (PCA) (Wen et al., 2017). PCA showed the distribution of origin data. The first principal component of variable importance in the projection (VIP) was obtained. The VIP values summarize the contribution of each variable to the model. The metabolites with VIP > 1 and *p* < 0.05 (student *t*-test) were considered as significantly altered metabolites. In addition, commercial databases including KEGG^3^ and MetaboAnalyst^4^ were utilized to search the pathways of metabolites.

### Strain Isolation, Identification, and Greenhouse Experiment

The fresh cotton plants were sampled for the isolation of endophytic bacteria. The method used to isolate the endophytic bacteria was reported previously (Zhang et al., 2017). First, the surface of the cotton plants was sterilized with 75% ethanol for 30 s, immersed in 2% sodium hypochlorite for 3 min, and rinsed four times with sterile distilled water. Then, 3 g of surface-sterilized cotton plants was collected and ground in 5 mL of sterilized water. The resultant suspension was plated on Luria-Bertani (LB, 10 g/L tryptone, 5 g/L yeast extract, and 10 g/L NaCl) and King’s B (KB, 20 g/L protease peptone No. 3, 1.5 g/L K_2_HPO_4_, 1.5 g/L MgSO_4_⋅7H_2_O, and 10% glycerol) plate after 10 × dilution. The plates were then incubated at 30°C for 2 days. Colonies of different morphology were transferred onto new LB and KB plates for further characterization. All isolates were purified and stored at –80°C in a nutrient broth containing 15% glycerol. DNA extraction, amplification, and sequencing were performed as described by a previous study (Zhao H. et al., 2019; Zhuang et al., 2021). The antagonistic activity of the isolates against cotton *Verticillium* wilt was evaluated using the methods reported by Liu et al. (2021b). Briefly, spores of *V. dahliae* were diluted to 8 × 10^5^ mL^–1^ and 200 μL was inoculated on plates containing potato dextrose agar (PDA, 200 g/L potato and 20 g/L glucose). Then, the plates were permitted to dry under a laminar flow hood. Portions of cultures (3 μL) of strains were added to the center of plates, and the zone of inhibition was measured after incubation at 30°C for 4 days.

The greenhouse experiments also evaluated the capacity of *Pseudomonas* sp. to control cotton wilt disease. First, cotton seeds were sterilized in 1.5% NaOCl for 10 min with gentle shaking and washed four times with ddH_2_O. Subsequently, the sterilized seeds soaked in the bacterial suspension (28°C, 200 rpm for 12 h) at room temperature for 2 h and seeds soaked in LB or water were used as the control samples. Then, one seed was sown in each pot (the ratio of sterilized nutrient soil to vermiculite is 3:1). Six pots were used for each replicate, and three replicates were used for each isolated bacterial strain. The cotton plants were placed in the growth chamber at 28°C with 12-h light/12-h dark conditions. When the second euphylla appeared, 10 mL spores of *V. dahliae* (1 × 10^8^mL^–1^) were added to the soil of the cotton plant, and this process was repeated every 2 days. In total, 30 mL spores of *V. dahliae* were added. The disease index of cotton plants was calculated after 40 days. Cotton plants were checked for severity based on the disease index described previously (Gu et al., 2021). Briefly, we rated the disease severity on a scale from 0 to 6 according to the percentage of symptoms. The score of 0 indicated no visible symptoms, and score of 1, 2, 3, 4, 5, or 6 described 0–10%, 11–25%, 26–50%, 51-75%, 76–90%, and >90%, discoloration or defoliation, respectively. The disease index was calculated as follows:

Disease index = [Σ(Rating × Number of diseased plants)/Total number of plants × Highest rating] × 100

## Results

### Assembly Patterns of the Healthy and Diseased Cotton Plants

The endophytic bacterial community of cotton plants was characterized by Illumina HiSeq sequencing. In total, 803,958 high-quality sequences were obtained, and each sample contained between 65,586 and 68,096 (66,997 ± 643) reads. All the sequences were clustered into 700 operational taxonomic units (OTUs) with > 97% similarity. Meantime, we also checked the disease index of different cultivars, and the results showed that the disease index of susceptible cultivars was significantly higher than that of the resistant cultivars in the experimental area (unpaired *t-*test, *P* < 0.001) (Supplementary Figure 2).

**FIGURE 1:**
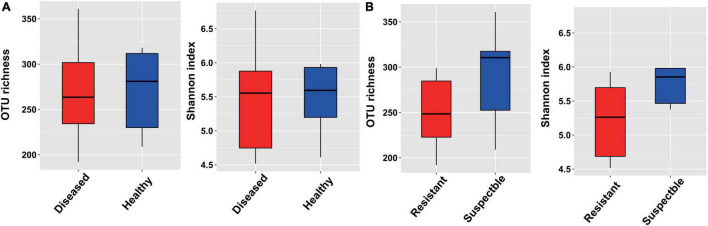
The alpha-diversity measurements based on the bacterial endophytic bacterial community. **(A)** For different healthy conditions. **(B)** For different cultivars.

Alpha-diversity of healthy and diseased cotton plants was measured by Shannon and observed OTU indices. It showed that the alpha-diversity values of healthy plants were higher than those of the diseased plants (Figure 1A). Meanwhile, we also compared the alpha-diversity of different cultivars and found that the alpha-diversity values of susceptible cultivars were higher than those of the resistant cultivars (Figure 1B). Subsequently, the samples were divided by cultivars and analyzed separately. The results displayed that the values of healthy plants were higher than those of the diseased plants for resistant cultivars. However, the index of diseased plants was higher than that of the healthy plants for susceptible plants, but the difference was not found to be significant (Supplementary Figure 3).

### Co-occurrence Patterns of Bacterial Community of Healthy and Diseased Cotton Plants

**FIGURE 2:**
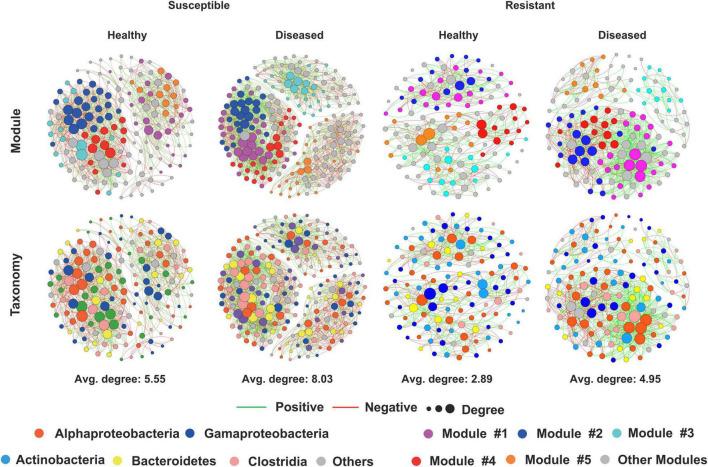
Network visualization of the interaction architecture in bacterial community of healthy and diseased cotton plants.

To investigate how cotton *Verticillium* wilt affects the co-occurrence patterns of the cotton endophytic community, we analyzed the bacterial community network of healthy and diseased plants. Based on the network analysis, we found that the healthy conditions showed an effect on network complexity. The average degree was used to assess the network complexity and showed that the endophytic bacterial community of the diseased plants (8.03 and 4.95) was more complex than that of the healthy plants (5.55 and 2.89, Figure 2). Additionally, the results also displayed that the susceptible cultivars possessed a higher degree than the resistant cultivars in healthy and diseased conditions. Furthermore, the number of “hub nodes” [nodes with high values of degree (>30) and closeness centrality (>0.6) in the network] from the diseased samples was higher than that of the healthy plants (Table 1). The taxonomic composition of the networks differed between healthy and diseased cotton plants, with most nodes annotated to Clostridia for diseased plants and Gammaproteobacteria for healthy plants of susceptible cultivars (Figure 2). Moreover, we recorded a higher ratio of positive to negative correlations in diseased cotton plants for susceptible and resistant cultivars. In addition, a higher average path distance was found in the healthy cotton plants (Table 1).

**TABLE 1:** Topological properties of the co-occurrence networks of healthy and diseased cotton plants.

Category	Average degree	Node	Edge	Positive edge	Negative edge	Ratio of positive to negative correlations	Modularity	Average clustering coefficient	Average path distance	Hub node
**Susceptible cotton**										
Healthy	5.55	153	849	500	349	1.43	0.845	0.001	2.059	2
Diseased	8.03	216	1734	1246	488	2.55	0.855	0	1.989	7
**Resistant cotton**										
Healthy	2.89	124	358	192	166	1.16	0.708	0	2.118	0
Diseased	4.95	128	633	412	221	1.86	0.847	0	2.079	4

### Differences in Endophytic Bacterial Community Between Healthy and Diseased Cotton Plants

**FIGURE 3:**
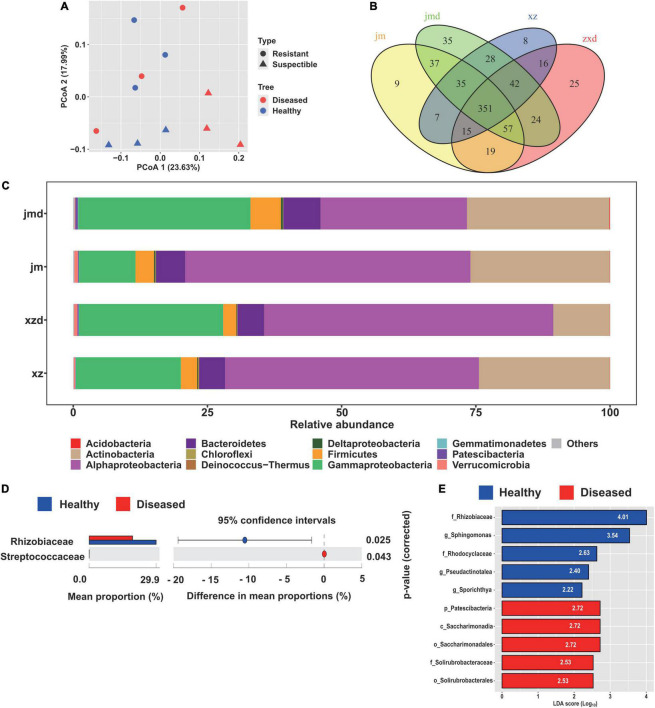
Comparative analysis of the endophytic bacterial community under different healthy conditions. **(A)** Unconstrained principal coordinate analysis (PCoA) of beta-diversity using Bray-Curtis distances of the bacterial community in healthy and diseased samples. **(B)** Venn diagrams showing the shared and specific bacterial OTUs among the healthy and diseased samples of different cultivars. **(C)** Relative abundance of the abundant bacterial taxa in healthy and diseased cotton plants. **(D)** Differential abundance analysis of bacterial OTUs in healthy and diseased samples, and Welch’s test was performed to calculate the significance of differences at the family level. **(E)** The biomarker taxa of bacterial communities among healthy and diseased cotton plants. Only the top five most specific biomarker taxonomies are displayed.

The principal coordinate analysis (PCoA) plot of microbial communities revealed a clear separation between the cotton plants of different cultivars. The two main coordinates extracted explained 41.62% of the variation, of which PC1 explained 23.63% of the variation and PC2 explained 17.99% of the variation (Figure 3A). Moreover, the cultivars had a significant influence on the endophytic bacterial community (*R*^2^ = 14.98%, *P* < 0.05). Furthermore, hierarchical clustering analysis revealed a separate clustering between healthy and diseased plants (Supplementary Figure 4). The results also showed that the healthy conditions had a strong effect on the bacterial endophytic community.

The bacterial composition at the phylum level was analyzed in the cotton samples. We found that the most abundant bacterial phyla were Alphaproteobacteria (45.4%), Gammaproteobacteria (22.3%), Actinobacteria (21.8%), Bacteroidetes (5.5%), and Firmicutes (3.7%) (Figure 3C). In addition, taxonomic classification showed that *Rhizobiaceae* was the most abundant bacteria in healthy and diseased cotton plants at the family level. Furthermore, we found that the population of *Rhizobiaceae* was significantly increased in the healthy cotton plants at the family level, whereas *Streptococcaceae* was significantly decreased in the healthy cotton plants compared with diseased cotton plants (Figure 3D). Meanwhile, the LEfSe results showed that *Rhizobiaceae* and Patescibacteria were the biomarker taxa for healthy and diseased cotton plants (Figure 3E).

To determine which OTUs are responsible for the healthy conditions and cultivar effect, we performed OTU analysis with OTUs identified from healthy and diseased plants, and different cultivars (Figure 3B). In the resistant cultivars, we identified 8 and 25 OTUs that were specifically observed in healthy and diseased plants, respectively. The OTUs that were specifically noticed in healthy plants primarily correspond to Clostridia, Bacteroidia, Actinobacteria, and Verrucomicrobiae. In contrast, the diseased plants specifically showed the presence of Clostridia, Alphaproteobacteria, Gammaproteobacteria, Bacteroidia, Deinococci, and Negativicutes at the class level. For the susceptible cultivars, nine OTUs consisting of Clostridia, Bacteroidia, Gammaproteobacteria, Actinobacteria, and Verrucomicrobiae were specifically selected by healthy plants, and 35 OTUs belonging to Clostridia, Bacteroidia, Alphaproteobacteria, Deltaproteobacteria, Gammaproteobacteria, Acidimicrobiia, Nitrospira, Verrucomicrobiae, Nitriliruptoria, and Melainabacteria were specifically selected by diseased plants (Figure 3B and Supplementary Table 1). Furthermore, the Venn diagram analysis of all samples indicated that the diseased plants (84) had higher specific OTUs than the healthy plants (24). The OTUs selected by healthy plants annotated as Ruminococcaceae, Opitutaceae, Sphingobacteriaceae, Weeksellaceae, Microscillaceae, and Nocardioidaceae, while the OTUs selected by diseased plants belonged to Ruminococcaceae, Lachnospiraceae, Muribaculaceae, Burkholderiaceae, and Chitinophagaceae (Supplementary Figure 5A). In addition, we found that the susceptible cultivars (81) possessed more specific OTUs than the resistant cultivars (49). The 81 OTUs mainly corresponded to Ruminococcaceae, Muribaculaceae, Desulfovibrionaceae, Flavobacteriaceae, Burkholderiaceae, Xanthomonadaceae, Sphingobacteriaceae, and Chitinophagaceae, and the 49 OTUs mainly corresponded to Ruminococcaceae, Lachnospiraceae, Sphingobacteriaceae, and Weeksellaceae at the family level (Supplementary Figure 5B).

Remarkably, the different abundance analysis indicated that two OTUs which corresponded to Xanthomonadaceae and Saccharimonadaceae at the family level were significantly enriched in the diseased plants. In contrast, one OTU, belonging to *Pseudomonas*, was significantly depleted in the diseased plants. However, there were no enriched or depleted OTUs in the susceptible cultivars and all the samples (Supplementary Figures 5, 6). For the different cultivars, we found that three and six OTUs were significantly enriched in resistant and susceptible cultivars, respectively. In the resistant cultivars, the OTUs belonged to Burkholderiaceae, Moraxellaceae, and Opitutaceae at the family level, and in the susceptible cultivars, the OTUs corresponded to Pseudomonadaceae, Xanthomonadaceae, Cellvibrionaceae, and Ruminococcaceae (Supplementary Figure 5D).

### Root Exudate Profiles of the Resistant and Susceptible Cotton Cultivars

**FIGURE 4:**
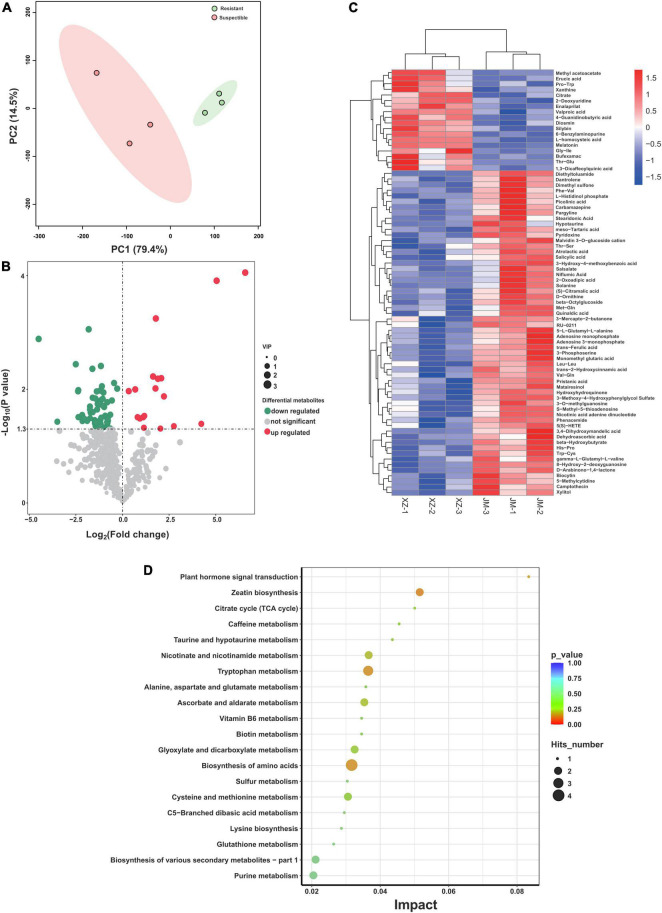
Analysis of exudation profiles between resistant and susceptible cotton cultivars. **(A)** PCA plot of the root exudate profiles of two cotton cultivars. **(B)** The comparison of volcano plots of differentially expressed metabolites between the resistant and susceptible cultivars. **(C)** Heatmap of identified differentially expressed metabolites from resistant and susceptible samples. **(D)** Pathway enrichment analysis of differential metabolites from resistant and susceptible cultivars. XZ is the resistant cultivar and JM is the susceptible cultivar.

The root exudates of resistant and susceptible cotton cultivars were analyzed by UHPLC-QE-MS after collecting from cotton root samples. A total of 3,777 peaks were detected, and 212 compounds were identified across all the samples, including lipids and lipid-like molecules, benzenoids, organic acids and derivatives, organic oxygen compounds, organoheterocyclic compounds, phenylpropanoids and polyketides, and nucleosides, nucleotides, and analogs at the superclass level. The overall exudation patterns of the resistant and susceptible cotton plants were found to be distinct. The first two components of the PCA explained 93.9% of the total variance (79.4 and 14.5% for PC1 and PC2, respectively), and this analysis clearly separated the susceptible and resistant samples (Figure 4A). Furthermore, the data revealed the presence of multiple metabolites that showed significant differences in abundance between susceptible and resistant cultivars according to the VIP thresholds (VIP > 1) and the student’s *t*-test (*P* < 0.05). A total of 76 compounds were found to be significantly different between these two cultivars. Specifically, 18 compounds showed a higher relative abundance in the resistant cultivars, and 58 compounds showed a higher relative abundance in the susceptible cultivars (Figure 4B). To further reveal the relative content of all the annotated and significantly differentially expressed metabolites in the two cultivars and determine their relationships, cluster analysis was conducted, and the results were presented as a heatmap (Figure 4C). The susceptible cultivars had more number of differentially expressed metabolites. Then, the pathway analysis based on the significantly different metabolites was carried out using the KEGG enrichment analysis. The results displayed that pathways related to plant hormone signal transduction (salicylic acid), biosynthesis of various secondary metabolites, biosynthesis and metabolism of amino acids, including tryptophan, alanine, aspartate, glutamate, cysteine, and methionine, and zeatin biosynthesis (Figure 4D) were significantly altered.

### *Pseudomonas* sp. Exhibited Stronger Inhibition to *Verticillium* Wilt of Cotton

**FIGURE 5:**
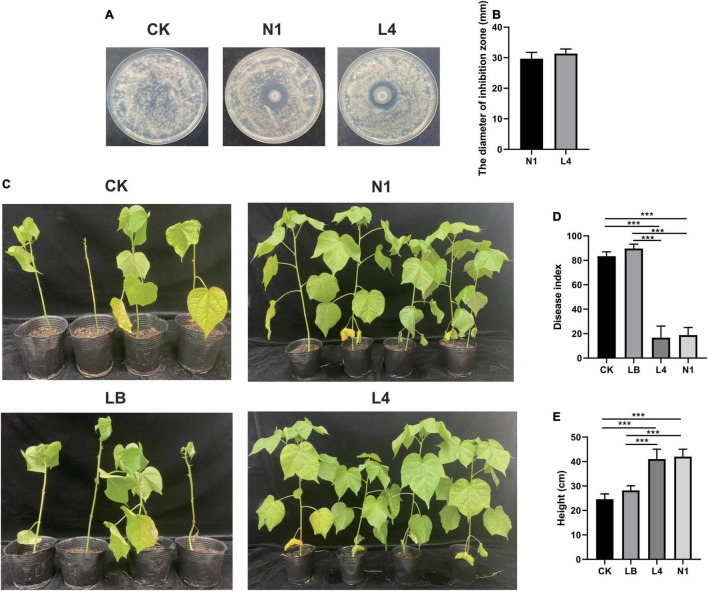
Effect of *Pseudomonas* sp. on the cotton wilt disease. **(A)** Effect of single bacterial strain (N1 and L4) on the *Verticillium* wilt of cotton. **(B)** Diameter of inhibition zone of single bacteria strain (N1 and L4). Error bars indicate ± SD of three replicates. **(C)** The phenotypes of cotton inoculated with single bacteria after 40 days of growth in a growth chamber. Effect of the single bacteria (N1 and L4) on the disease index **(D)** and the height of cotton plants **(E)**. *** Represents obvious differences at *P* < 0.001 compared with the control.

To further characterize the antagonist effect of *Pseudomonas* sp. on the cotton *Verticillium* wilt, we isolated 30 candidate bacteria from the healthy cotton plants. In total, two bacterial isolates were obtained and phylogenetically characterized based on the distinct 16S rDNA gene sequence, allowing us to recover strains for further studies. The results indicated that these two strains belonged to the genus *Pseudomonas* and inhibited the growth of cotton *Verticillium* wilt on the PDA plate, and the diameters of the inhibition zone were 29.67 ± 2.08 and 31.33 ± 1.53 for strains N1 and L4, respectively (Figures 5A,B). Furthermore, we were curious to know if the *Pseudomonas* sp. played a role in the control of the cotton wilt under greenhouse conditions. The results displayed that the disease index of cotton plants treated with the strains L4 and N1 was significantly lower than that of the control plants. There were no significant differences in the disease index between control and LB-treated plants (Figures 5C,D). Importantly, all inoculation treatments could promote plant growth. The height of plants treated with *Pseudomonas* sp. strains N1 and L4 was significantly higher than that of the control plants (Figure 5E). The results displayed that *Pseudomonas* sp. promoted the growth of cotton plants and protected the plants by inhibiting the growth of *V. dahlia*.

## Discussion

Here, the microbial community of healthy and diseased cotton plants, and the root exudate profiles of susceptible and resistant cultivars were surveyed using high-throughput sequencing and metabolomics analysis. The bacterial diversity and the composition of the bacterial community were affected by the cotton *Verticillium* wilt. Meanwhile, more differentially expressed metabolites were found in susceptible cultivars. Furthermore, the results showed that *Pseudomonas* strains were enriched in healthy cotton plants of resistant cultivars and inhibited the cotton *Verticillium* wilt. These findings provided a strategy for developing microbiome-based methods to control cotton *Verticillium* wilt.

### The Assembly Pattern of Diseased and Healthy Cotton Plants

The microbiome of the plant plays a crucial role in plant health. Microbial diversity and distinct changes in the microbial community were previously shown to be related to the disease incidence (Berg et al., 2017). In the current study, we found that the alpha-diversity of the diseased plants was higher than that of the healthy plants for the endophytic bacterial microbiome (Figure 1). The previous studies found that the bacterial diversities of infected root and stem samples were significantly higher than those of the healthy plants. The pathogen disrupted the plant defense system, which allowed entry to more number of organisms into the plant (Hu et al., 2020). Moreover, the results also showed that the infected samples had lower bacterial diversity compared to the healthy samples based on the healthy and decaying sugar beets (Kusstatscher et al., 2019; Liu et al., 2019). The decrease in the microbial diversity of plants was probably related to the invasion by the pathogenic species into the communities. The plant microbiome was found to be affected by many factors, such as soil properties, plant genotype, plant developmental stage, and background microbial composition of the soil (Qiao et al., 2017). We also found that the different cotton cultivars affected the composition of the bacterial microbiome (Figure 3A). Furthermore, the plant developmental stage had an impact on the microbial community. The richness indices of the bacterial species in the shoot and roots increased with the growth of the banana plant (Liu et al., 2019). The endosphere microbiome of cotton during plant growth and disease development should be further studied.

In addition, we found that the alpha-diversity in the susceptible cultivars was higher than that of the resistant cultivars regardless of the healthy conditions in the bacterial community, which is consistent with the findings in a previous study, which showed that cucumbers susceptible to *Fusarium oxysporum* f. sp. *cucumerinum* exhibited higher bacterial community evenness than the resistant variety of cucumber (Supplementary Figure 3, Wen et al., 2020). High diversity was observed in the susceptible cultivars, which could be attributed to a relatively high variety of exudates supporting more microbial niches (Wen et al., 2020). We also found that the susceptible cultivars had more different abundant compounds compared with the resistant cultivars (Figure 4). Furthermore, we displayed that the network complexity of diseased plants was more complex than that of healthy plants, and the results were consistent with the previous report (Hu et al., 2020, Figure 2). The pathogenic produce positive feedback that enhances their competitiveness and subsequent interactions with the neighbors by changing the composition. Importantly, the highly connected and modular microbiota could prime the plant immune system for accelerated activation of defense against the pathogen (Hu et al., 2020).

### Disease-Induced Changes in Endophytic Bacterial Microbiome Composition

Plants can alter their microbiome composition upon pathogen infection and recruit some disease-resistance and growth-promoting beneficial microbes for their survival. For instance, the relative abundances of *Pseudomonas*, *Bacillus*, and *Falsibacillus*, which are often considered to be plant-beneficial bacteria, showed a significant decrease in the diseased plants when compared to the healthy plants. These genera colonize different plant compartments and play a vital role in modulating host performance, particularly in the suppression of plant pathogens (Berendsen et al., 2018; Hu et al., 2020). In the present study, the comparative analysis of the composition of the healthy and diseased plants showed that the healthy plants were enriched in *Rhizobiaceae*, which is also the biomarker taxa of healthy plants, and diseased plants were enriched in *Streptococcaceae* (Figures 3D,E). Moreover, we found that the healthy plants were enriched in one OTU, annotated as *Pseudomonas*, and the diseased plants were enriched in two OTUs, classified as *Xanthomonadaceae* and *Saccharimonadaceae*, of resistant cultivars (Supplementary Figure 6). The enriched OTUs of healthy plants probably play an important role in protecting the plants from cotton *Verticillium* wilt. This finding is consistent with the previous report (Yuan et al., 2020; Zhang et al., 2021). Furthermore, the results displayed that the healthy plants and resistant cultivars contained specific OTUs corresponding to Sphingobacteriaceae, which included the biocontrol genus of *Sphingobacterium* (Xu et al., 2020). We also need to confirm the capable of suppression cotton *Verticillium* wilt. However, recent studies have reported that several potential beneficial bacteria, such as *Pseudomonas*, *Streptomyces*, and *Bacillus*, were enriched in diseased plants (Gao M. et al., 2021). The opposite results observed in different plant species need further exploration.

Deciphering the composition of the healthy and diseased plants is critical for harnessing the plant microbiome to enhance plant growth and health. The previous studies showed that Acidobacteria, Actinobacteria, Bacteroidetes, Planctomycetes, Proteobacteria, and Verrucomicrobia were the major taxa in the cotton rhizosphere (Qiao et al., 2017), while we found that Proteobacteria, Actinobacteria, Bacteroidetes, and Firmicutes were the main taxa in the cotton endophytic bacterial community (Figure 3C). There are some differences between rhizosphere and endophytic communities, and the crop microbiome is mainly derived from soil and is gradually enriched and filtered at different plant compartment niches (Xiong et al., 2021). The soil microorganisms likely affect the pathogen migration, and the interaction between plant endophytes and soil microorganisms facilitates the pathogen invasion process. It was found that pathogen invasion may begin in the bulk soils, then transfer to the plant roots, and further infect the plant stems, causing bacterial wilt by *Ralstonia* (Hu et al., 2020). Under stress conditions, a plant could attract distant beneficial microbes by actively releasing root exudates, such as amino acids, nucleotides, and long-chain organic acids. Based on the metabolism results, we found that the susceptible cultivars had more differentially expressed metabolic pathways (Figure 4B). Pathway enrichment analysis showed that differentially expressed metabolic pathways were related to plant hormone signal transduction, biosynthesis of various secondary metabolites, and biosynthesis and metabolism of amino acids (Figure 4D). Importantly, the amino acids could induce plant growth through rhizosphere bacteria (Zhao et al., 2019). The relationship between rhizosphere and root exudates needs further study.

### The Pathogen Growth Inhibition and Plant Disease Control

*Pseudomonas* sp. are widely used as biocontrol agents to combat plant diseases. In the previous study, *Pseudomonas* spp. could directly inhibit the growth of cotton *Verticillium* wilt under laboratory and greenhouse conditions. In the meantime, the abundances of *Pseudomonas* in the soil microbial community with less incidence had higher abundances change are higher (Erdogan and Benlioglu, 2010; Zhang et al., 2022). The *Pseudomonas* was the key plant growth-promoting rhizobacteria in the rhizosphere of garlic plants and had the ability to promote plant growth (Zhuang et al., 2021). The genera of *Bacillus* and *Pseudomonas* are the most dominant biocontrol strains in the market, and these two genera could coexist and cooperate with each other. The bio-organic fertilizers stimulate the increase of the *Pseudomonas* spp. in the soil microbiome to enhance plant disease suppression, and *B. velezensis* SQR9 induced the enrichment of *Pseudomonas* sp. in the rhizosphere and promoted the growth of cucumber plants synergistically (Tao C. et al., 2020; Yin et al., 2021; Sun et al., 2022). Moreover, the cotton rhizosphere soil was temporarily altered after applying *B. axarquiensis* strain. Among them, *Streptococcus* and *Pontibacter* were decreased, and *Lysobacter* and *Bacillus* were significantly increased (Gao C. et al., 2021).

In the present study, we found that two *Pseudomonas* strains could inhibit the cotton *Verticillium* wilt under the laboratory and greenhouse conditions (Figure 5). However, it has been reported that complex microbial community assemblages rather than single microbial strains protected plants from pathogen infection based on multiomics sequencing technologies. More number of studies revealed that the synthetic communities could be applied to achieve sustainable agriculture. The seven microbiome strains together showed better protection of maize roots against *Fusarium verticillioides* infection than the individual strains (Niu et al., 2017). The members of *Chitinophagaceae* and *Flavobacteriaceae* were enriched in the plant endosphere following pathogen invasion, and reconstruction of a synthetic community of *Flavobacterium* and *Chitinophaga* consistently suppresses fungal root disease against sugar beet *Rhizoctonia* damping-off disease (Carrion et al., 2019). Importantly, the enriched bacteria acted as an early warning agent by activating the salicylic acid and jasmonic acid pathways in the presence of the pathogen (Liu et al., 2021a). Also, the results revealed that beneficial bacteria showing high abundance and low abundance performed different functions. The high-abundance bacteria mainly protected the plants by promoting plant growth and inhibiting pathogen invasion, while the low-abundance bacteria controlled the diseases by inducing resistant systems (Ritpitakphong et al., 2016; Jack and Nelson, 2018; Li et al., 2021). *Pseudomonas* spp. could colonize the roots of plants and produce antibiotics to control the disease (Tao X. et al., 2020). Moreover, the endophyte *P. fluorescens* PICF7 could trigger defense responses and compete in the niche with the pathogen in roots (Prieto et al., 2009; Gómez-Lama Cabanás et al., 2014). In this study, it displayed strong inhibition of cotton *Verticillium* wilt when the cotton plant was treated with isolated *Pseudomonas* strains, and it also promoted the plant growth (Figure 5). We further need to confirm the mechanism by which isolated *Pseudomonas* strains prevent the cotton *Verticillium* wilt.

## Conclusion

In this study, we provide comprehensive evidence that the cotton *Verticillium* wilt affects the endophytic microbiome based on the sequencing method. In particular, the healthy plants could recruit beneficial bacteria, such as *Pseudomonas*, to suppress the cotton *Verticillium* wilt. Further investigations showed that the isolated *Pseudomonas* strains could inhibit the cotton *Verticillium* wilt. These findings suggest that the endophyte could develop microbiome-based solutions for sustainable crop production. However, additional studies are needed to confirm the obtained results, owing to the fact that the study was performed in the late growth stage of cotton in Korla.

## Data Availability Statement

The datasets presented in this study can be found in online repositories. The names of the repository/repositories and accession number(s) can be found below: NCBI (accession: PRJNA820148).

## Author Contributions

YD and HL designed the experiment. QZ and XM analyzed the data and wrote the manuscript. HL collected the samples from the field. All authors approved the manuscript.

## Conflict of Interest

The authors declare that the research was conducted in the absence of any commercial or financial relationships that could be construed as a potential conflict of interest.

## Publisher’s Note

All claims expressed in this article are solely those of the authors and do not necessarily represent those of their affiliated organizations, or those of the publisher, the editors and the reviewers. Any product that may be evaluated in this article, or claim that may be made by its manufacturer, is not guaranteed or endorsed by the publisher.

## Footnotes

1
https://cloud.oebiotech.cn

2
http://huttenhower.sph.harvard.edu/galaxy/

3
http://www.kegg.jp

4
http://www.metaboanalyst.ca/

## Supplementary Material

Supplementary Figure 1 The experimental design and symptoms of the diseased and healthy plants. **(A)** The process adopted to construct the experimental field. **(B)** The picture of the cotton plant from the experimental area. **(C)** The infected plants were found in the field. **(D)** The symptoms of the diseased and healthy stems of the cotton plant. **(E)** The culture isolates were obtained from the diseased plants.

Supplementary Figure 2 The disease index of susceptible and resistant cultivars in the field experiment. Values are representative of three plots. The analysis was performed using a *t*-test. ^∗∗∗^*P* < 0.001.

Supplementary Figure 3 The comparative analysis of alpha-diversity indices. **(A)** The observed and Shannon index of different healthy conditions divided by cultivars. **(B)** The observed and Shannon index of different cultivars divided by plant healthy conditions.

Supplementary Figure 4 Hierarchical clustering of the bacterial samples based on Bray–Curtis dissimilarity. Samples were clustered according to the complete method.

Supplementary Figure 5 The specific and common OTUs between different healthy conditions and cultivars. **(A)** The different healthy conditions. **(B)** The different cultivars. **(C)** The enriched OTUs in healthy and diseased plants based on all samples. **(D)** The enriched OTUs in susceptible and resistant cultivars based on all samples.

Supplementary Figure 6 The volcano plot illustrating the enriched OTUs for resistant and susceptible samples. Each point represents a single OTU. Each red point represents an individual enriched OTU for diseased plants and each blue point represents an individual enriched OTU for healthy plants.

Supplementary Table 1 The specific OTUs for healthy and diseased samples of different cultivars.
