# Supplementary figures and images for: Pseudomonas spp. Enriched in Endophytic Community of Healthy Cotton Plants Inhibit Cotton Verticillium Wilt
Source: Front Microbiol. 2022 Jul 18;13:906732. doi: 10.3389/fmicb.2022.906732 (PMC9339998; doi:10.3389/fmicb.2022.906732)

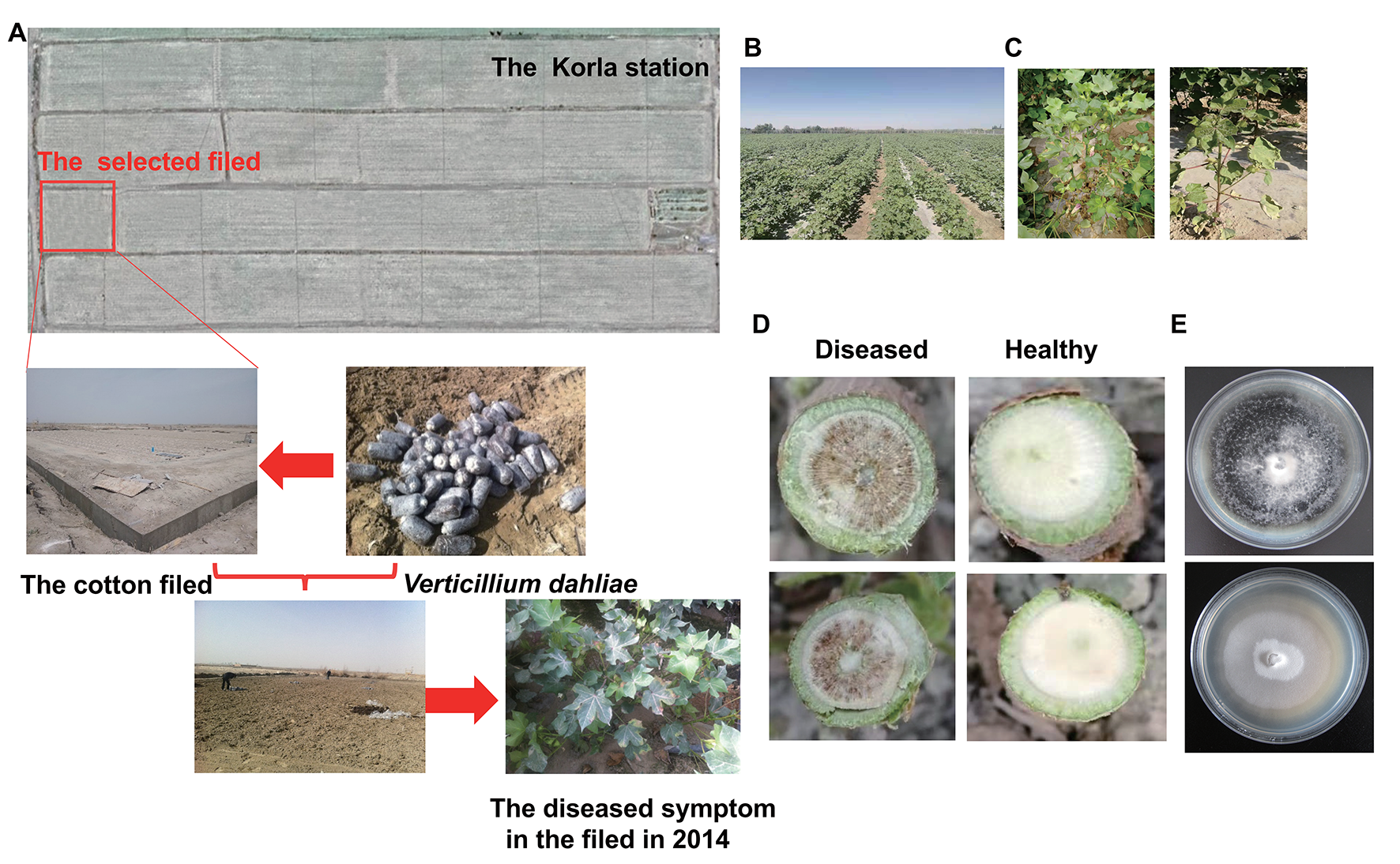

Supplement: Supplementary Figure 1 — The experimental design and symptoms of the diseased and healthy plants. (A) The process adopted to construct the experimental field. (B) The picture of the cotton plant from the experimental area. (C) The infected plants were found in the field. (D) The symptoms of the diseased and healthy stems of the cotton plant. (E) The culture isolates were obtained from the diseased plants. [file Image_1.TIF]

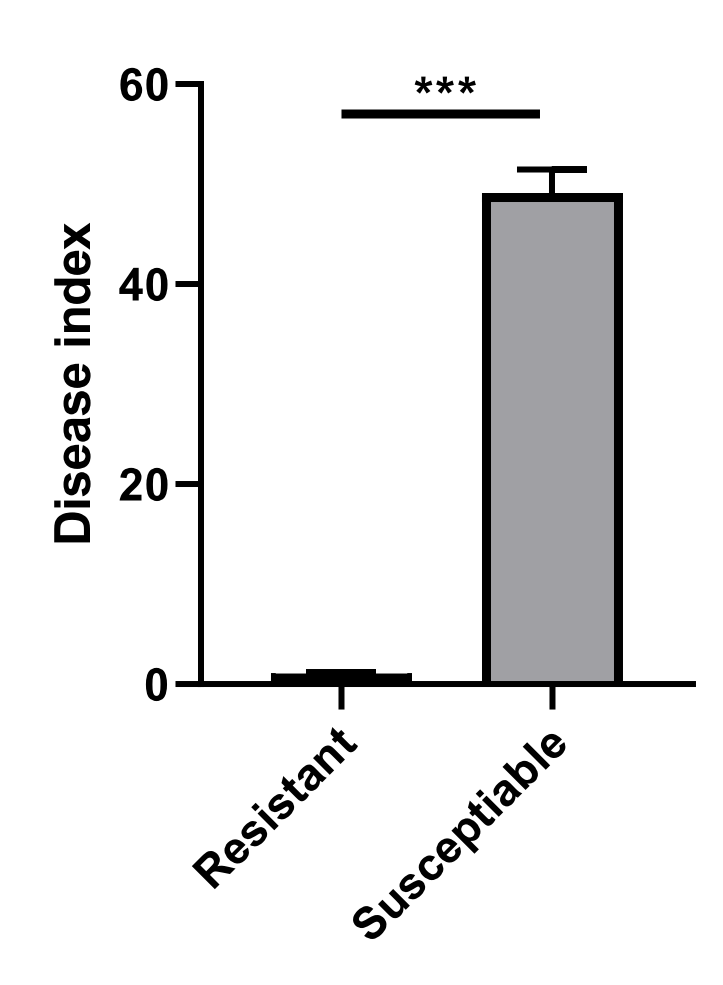

Supplement: Supplementary Figure 2 — The disease index of susceptible and resistant cultivars in the field experiment. Values are representative of three plots. The analysis was performed using a t-test. ∗∗∗P < 0.001. [file Image_2.TIF]

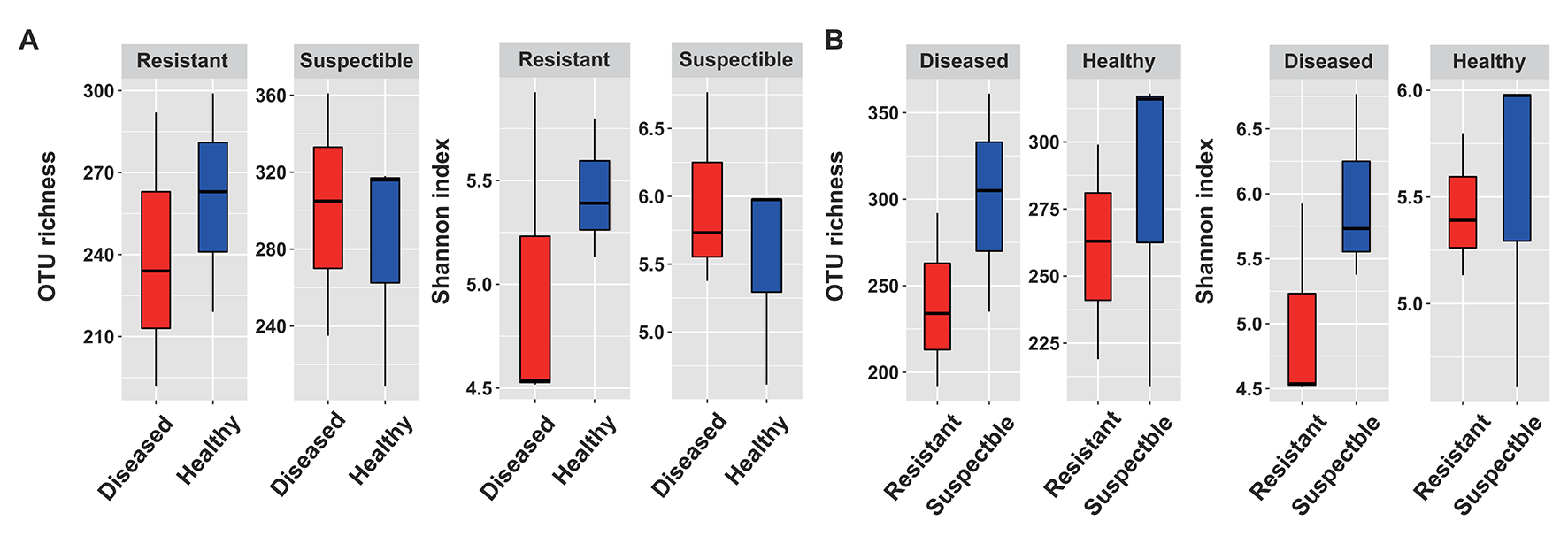

Supplement: Supplementary Figure 3 — The comparative analysis of alpha-diversity indices. (A) The observed and Shannon index of different healthy conditions divided by cultivars. (B) The observed and Shannon index of different cultivars divided by plant healthy conditions. [file Image_3.TIF]

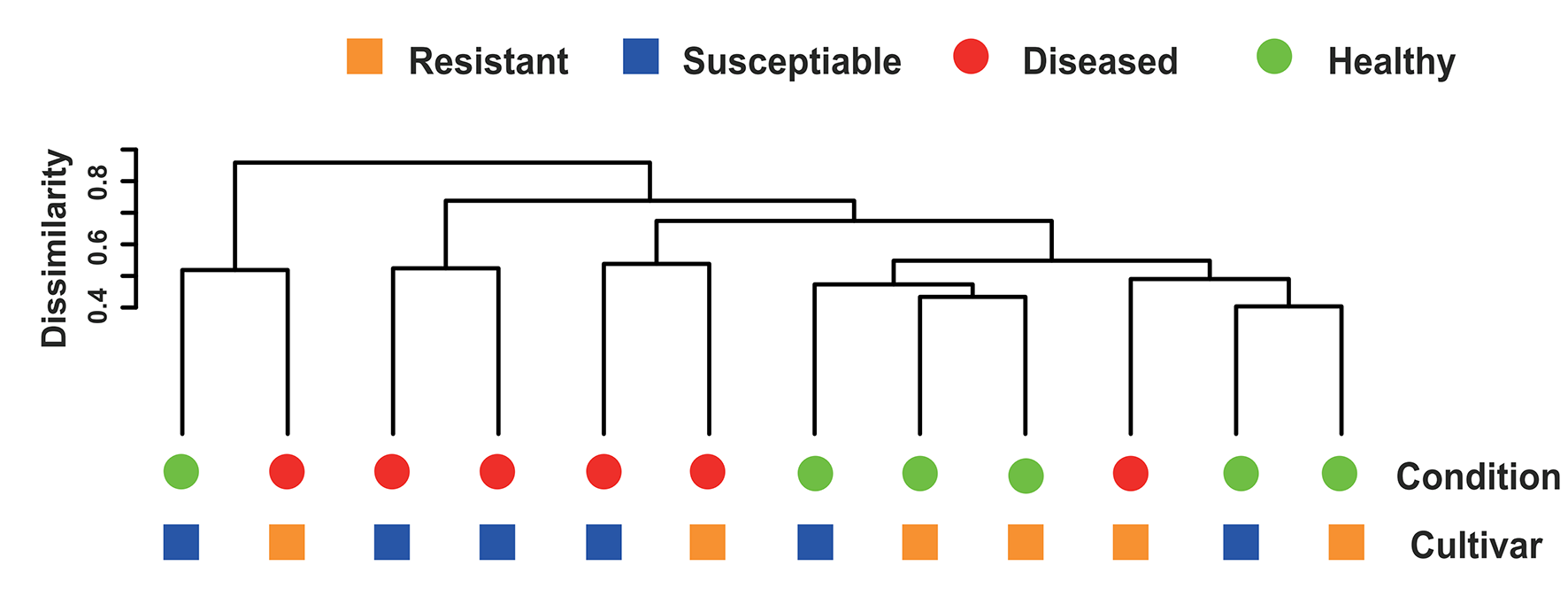

Supplement: Supplementary Figure 4 — Hierarchical clustering of the bacterial samples based on Bray–Curtis dissimilarity. Samples were clustered according to the complete method. [file Image_4.TIF]

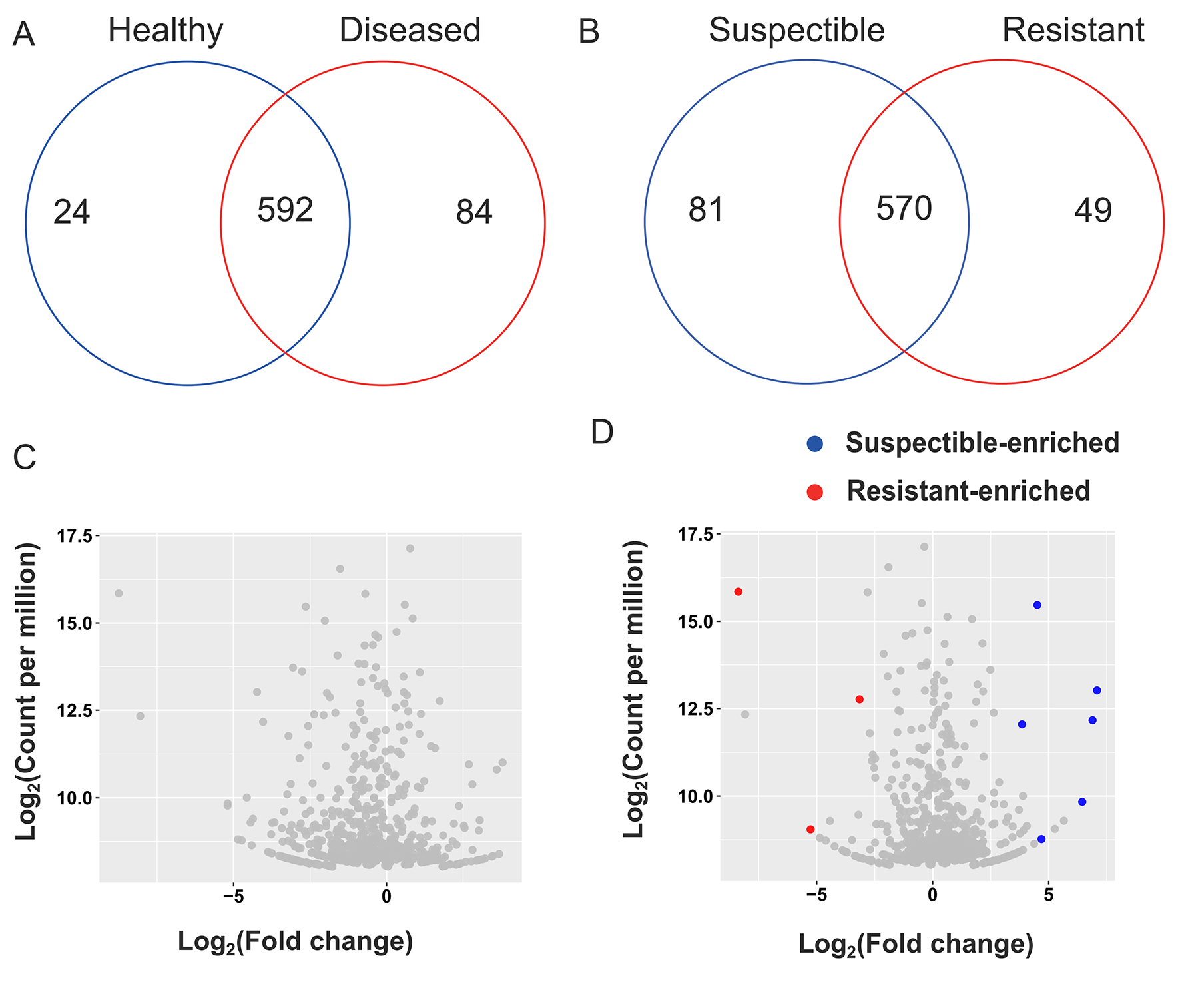

Supplement: Supplementary Figure 5 — The specific and common OTUs between different healthy conditions and cultivars. (A) The different healthy conditions. (B) The different cultivars. (C) The enriched OTUs in healthy and diseased plants based on all samples. (D) The enriched OTUs in susceptible and resistant cultivars based on all samples. [file Image_5.TIF]

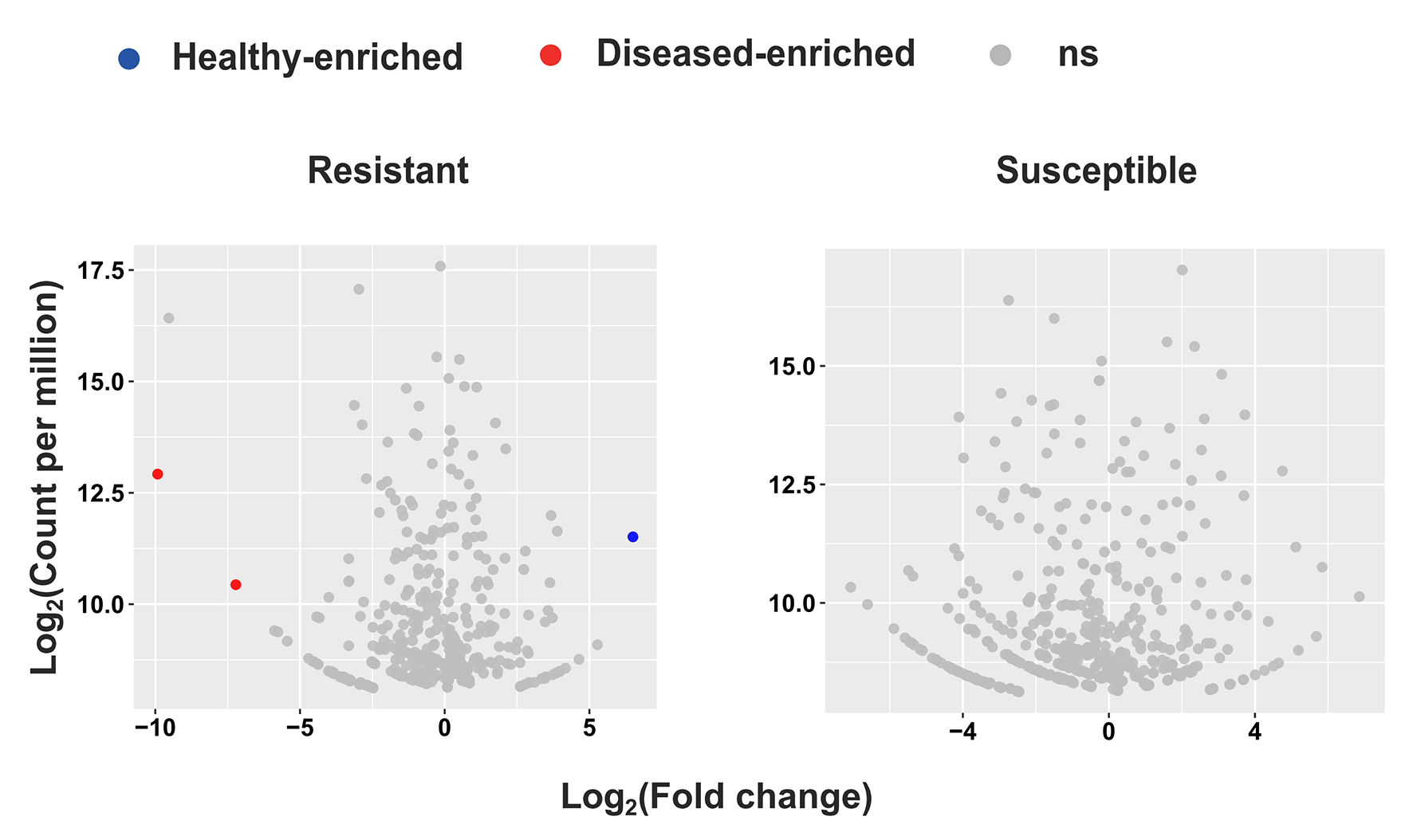

Supplement: Supplementary Figure 6 — The volcano plot illustrating the enriched OTUs for resistant and susceptible samples. Each point represents a single OTU. Each red point represents an individual enriched OTU for diseased plants and each blue point represents an individual enriched OTU for healthy plants. [file Image_6.TIF]
